# Role of Connexin 43 phosphorylation on Serine-368 by PKC in cardiac function and disease

**DOI:** 10.3389/fcvm.2022.1080131

**Published:** 2023-01-12

**Authors:** Renju Pun, Michael H. Kim, Brian J. North

**Affiliations:** ^1^Department of Biomedical Sciences, School of Medicine, Creighton University, Omaha, NE, United States; ^2^CHI Health Heart Institute, School of Medicine, Creighton University, Omaha, NE, United States

**Keywords:** gap junctions, Connexin 43, phosphorylation, protein kinase C, cardiology, cardiac disease

## Abstract

Intercellular communication mediated by gap junction channels and hemichannels composed of Connexin 43 (Cx43) is vital for the propagation of electrical impulses through cardiomyocytes. The carboxyl terminal tail of Cx43 undergoes various post-translational modifications including phosphorylation of its Serine-368 (S368) residue. Protein Kinase C isozymes directly phosphorylate S368 to alter Cx43 function and stability through inducing conformational changes affecting channel permeability or promoting internalization and degradation to reduce intercellular communication between cardiomyocytes. Recent studies have implicated this PKC/Cx43-pS368 circuit in several cardiac-associated diseases. In this review, we describe the molecular and cellular basis of PKC-mediated Cx43 phosphorylation and discuss the implications of Cx43 S368 phosphorylation in the context of various cardiac diseases, such as cardiomyopathy, as well as the therapeutic potential of targeting this pathway.

## Introduction

Cell–Cell communication is a well-established process in multicellular organisms and includes mechanisms such as endocrine, paracrine, and autocrine pathways. Gap junctions, composed of connexin proteins, are channels formed on the plasma membranes that can form a contiguous pore between adjacent cells to allow for exchange of ions, metabolic coupling, and electrical impulse propagation (1–3). The connexin gene family consists of 20 members in mice and 21 in humans (4) and are structurally characterized as transmembrane proteins with 9 domains: intercellular N- and C-terminal tails, 4 transmembrane domains (M1–M4), 2 extracellular domains (E1), and one cytoplasmic loop (CL) (1, 5). The amino acid sequences of the transmembrane domains and extracellular loops between the different family members are highly conserved whereas the amino-terminal domain and cytoplasmic loop show modest conservation. However, the C-terminal tails show little to no conservation, suggesting that they may be subjected to connexin specific regulatory mechanisms (6). The diversity of this domain among connexin family members also includes varying lengths of the C-terminal tail resulting in a range of molecular weights for the various proteins from 23 to 62 kDa. The nomenclature of connexins genes/proteins correspond to their molecular weight. For example, Cx43 and Cx32 have molecular weights of 43 and 32 kDa, respectively (7, 8).

Six connexins can oligomerize to form a hexamer or connexon in the plasma membrane leading to the formation of a hemichannel (9–11). In order to form a gap junction between two cells, hemichannels from adjacent cells dock in a head-to-head manner. Such assembly can be homotypic or heterotypic depending on the connexin composition of docking connexons, which themselves can be homomeric or heteromeric depending on the oligomerization of identical and different connexin proteins, respectively (12). This variability in the composition of these gap junctions affects the permeability and conductance between adjacent cells (13, 14). While genes encoding the various connexins are scattered throughout the genome, most connexins have a similar gene structure consisting of a single intron flanked by two exons (15, 16). The biosynthesis of connexins occurs on ribosomes found associated with the rough endoplasmic reticulum where the connexin coding mRNA gets translated directly into the endoplasmic reticulum membrane. As the connexin passes through the Golgi stacks, they oligomerize into a connexon which is trafficked along microtubules within the Golgi vesicle membrane system. Connexon-containing transport vesicles bud from the *trans*-Golgi and subsequently fuse with the plasma membrane, where they can remain as hemichannels on the plasma membrane or dock to connexons from adjacent cells to form a gap junction (17–19). The nexus region of cell-cell attachment consists of gap junction plaques and their associated proteins while the undocked hemichannels are found in a region surrounding the gap junction plaques termed the perinexus (20, 21). The degradation of connexins is initiated by the internalization of the connexon plaques into cytoplasmic annular junctions (22–24). Each gap junction has a short half-life of a few hours (25, 26), and once internalized connexons are disassembled into connexins that undergo degradation via either proteasomal or lysosomal pathways (27–29).

Connexin proteins differ in their expression patterns across tissues and organs (Table 1). Cx46 and Cx50 are abundantly expressed in the eye lens fiber cells while Cx26, Cx30, and Cx31 are prominently expressed in the organ of Corti within the inner ear (30–32). Mammalian cardiomyocytes express Cx40, Cx43, and Cx45 and the expression pattern of these three isoforms varies within the heart. While connexin isoform expression may differ between species, mice and human atrial cardiomyocytes express Cx43, Cx45, and Cx40 while ventricular cardiomyocytes express Cx43 and low levels of Cx45 (33–39). The Sino atrial and atrioventricular nodes express Cx45, Cx40, as well as Cx30.2 (33, 35, 37, 40); the His bundle and upper bundle branch express Cx40 and Cx45; whereas the lower bundle branch and Purkinje fibers express Cx40, Cx43, and Cx45 (35, 37, 41–44). Therefore, connexins exert influence within various tissues and organs, and their dysregulation or mutation promotes tissue-specific disease states. For instance, mutations in the *Cx30* gene lead to non-syndromic hearing loss, keratitis ichthyosis deafness (KID), a rare disorder with hearing impairment and rough skin plaques (45, 46). Similarly, mutations in *Cx26* are associated with 50% of non-syndromic hearing loss (47, 48). Cataracts in the eye lens are associated with mutations in *Cx46*, while mutations in the *GJA1* gene which encodes Cx43 lead to a rare autosomal syndrome called oculodentodigital dysplasia (ODD) in which patients display diverse phenotypes such as bone malformations, vision loss, and hypotrichosis (49, 50). Mutations in Cx43 can also cause several skin disorders such as congenital alopecia-1, eczema, and palmoplantar keratoderma (51). Other diseases that have been tied to connexin mutations include Alzheimer’s disease and osteoarthritis (52, 53). The expression levels of connexin isoforms differ between organs and the diseases associated with various isoforms are summarized in Table 1.

**TABLE 1:** Prevalence of connexin isoforms in various organs and their expression levels in diseases associated with the organs.

	Disease	Connexin expression	References
Heart 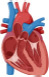	Atrial fibrillation	↓ Cx43	(237)
	Atherosclerosis	↑ Cx43	(238)
	Chronic myocardial infarction	↓ Cx43	(239)
Eye 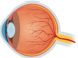	Retinoblastoma	↑ Cx37	(240)
	Epithelioid melanoma	↑ Cx43	(240)
	Diabetic retinopathy	↓ Cx43	(241)
Inner ear 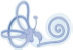	Noise induced hearing loss	↓ Cx26 ↓ Cx30	(242)
	Age related hearing loss	↓ Cx26 ↓ Cx30	(243)
Bones 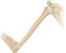	Oculodentodigital dysplasia (ODDD)	↓ Cx43	(244)
	Rheumatoid arthritis	↑ Cx43	(245)
Skin 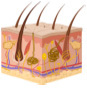	Acute eczema	↓ Cx43	(246)
	Chronic eczema	↑ Cx43	(246)
	Melanoma	↑ Cx26, Cx30.2 ↓ Cx43	(247)
Brain 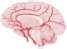	Alzheimer’s disease	↑ Cx43, Cx30 ↓ Cx47	(248)

Cx43 is the most abundantly expressed isoform in the heart. It is localized to the intercalated discs between atrial and ventricular myocytes and connects adjacent cardiomyocytes (54, 55). Mutations in Cx43 have been implicated in myocardial ischemia, cardiomyopathy, and heart failure (56–58). The C-terminal tail of Cx43, comprised of amino acids 232–382, has been extensively studied as it is subjected to various post-translational modifications such as phosphorylation, acetylation, *S*-nitrosylation, ubiquitination, and SUMOylation (17, 27, 59–65). Multiple serine and tyrosine residues within the Cx43 C-terminal tail are targeted by kinases such as Src, MAPK, and PKC (66, 67), and phosphorylation of these residues plays a key role in regulating the trafficking, assembly, permeability, and disassembly of gap junctions.

**FIGURE 1:**
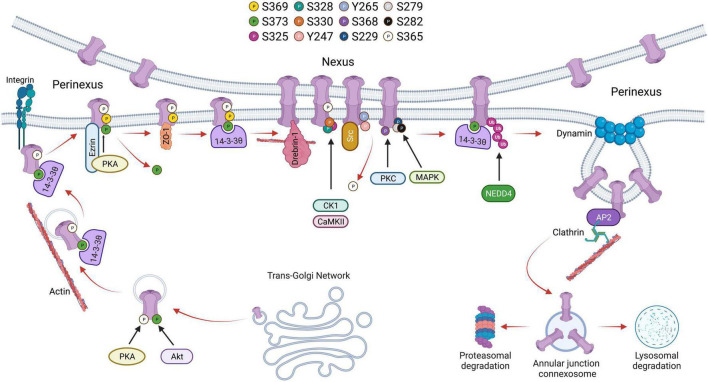
Schematic showing the life cycle of Cx43 beginning from trafficking through *trans*-Golgi network to accrual at the perinexus followed by gap junction activity at the nexus and degradation through endocytosis followed by proteasomal or lysosomal degradation. Phosphorylation of various serine residues in Cx43 is shown.

Cx43 containing vesicles bring newly formed hemichannels to the plasma membrane after exiting the *trans*-Golgi network during which Cx43 is phosphorylated on Serine-373 by Akt and Serine-365 by PKA (68, 69). 14–3–3 theta recognizes phosphorylated S373 and facilitates the delivery of hemichannels to the plasma membrane by tethering the hemichannels to integrin α5 (70). 14–3–3 theta is an isoform of the 14–3–3 adapter protein family that functions as critical regulators of a wide range of cellular processes (71, 72). Cx43 hemichannels then dock in the perinexus and translocate into the gap junction plaque (nexus) proper. Older channels are internalized and degraded from the central region of the gap junction plaque (26, 73, 74). Trafficking of Cx43 within the plasma membrane is also regulated by C-terminal tail phosphorylation. For instance, phosphorylation of Serine-369 by Protein kinase A (PKA) upregulates hemichannel aggregation in the perinexus. This is aided by the PKA associated protein Ezrin which binds to Cx43 forming a complex bringing PKA and the Cx43 C-terminal tail in close proximity to facilitate phosphorylation at S369 (75). This interaction is thought to precede ZO-1 association with the C-terminal tail of Cx43, which occurs following S373 dephosphorylation (76). ZO-1 is a cytoskeleton binding protein which docks with hemichannels in the periphery of gap junction plaques, scaffolding channels in the perinexus. 14–3–3 theta will again bind Cx43 hemichannels and facilitate their translocation to the nexus. Thus, phosphorylation of S373 is thought to be a trigger that induces the binding of 14–3–3 theta to Cx43 promoting channel translocation from the periphery of the gap junction into the plaque proper (77). As hemichannels of adjacent cells align, the gap junction plaque is stabilized by β-tubulin and Drebrin-1, which binds actin (78). A functional hemichannel is phosphorylated at multiple serine residues including Serine-325, Serine-328, and Serine-330 which are targeted by the kinases CaMKII and CK1 (79). Channel closure is regulated by phosphorylation at Tyrosine-247 and Tyrosine-265 initiated by the kinase Src. This phosphorylation is followed by PKC-mediated phosphorylation of S368 leading to a reduction in channel permeability (80). Interestingly, S368 phosphorylation occurs only after S365 dephosphorylation suggesting that S365 phosphorylation is a gatekeeper preventing downregulation of Cx43 by PKC (81). Src activity indirectly promotes phosphorylation of other serine sites including S373 by Akt and S225, S279, and S282 by MAPK, which leads to the recruitment of E3 ubiquitin ligase NEDD4 (82). Phosphorylated S279/282 increases the affinity of NEDD4 to the C-terminal tail of Cx43 by twofold (83). Ubiquitination of Cx43 allows for proteins such as Tsg101 and AP2, which are involved in clathrin-mediated endocytosis, to bind to the C-terminal tail of Cx43 (84, 85). During endocytosis, Dynamin serves to initiate scissoring of the gap junction bud and the formation of an annular gap junction (86). Translocation of the annular gap junction from the cell membrane to the cytoplasm is driven by Myosin VI (87) upon which it is subsequently degraded through the proteasomal and lysosomal pathways (88). The Cx43 lifecycle, and the role of C-terminal tail phosphorylation events, is outlined in Figure 1.

Amongst the many C-terminal residues targeted for phosphorylation, S368 has been the subject of extensive characterization with a particular emphasis on its role in the heart. For instance, phosphorylation of Cx43 S368 is necessary for myocardial conduction particularly during stress conditions such as metabolic stress (89). Therefore, the remainder of this review will focus on the relationship between Cx43 S368 phosphorylation by PKC isoforms and its consequences in cardiac health and disease.

## Cx43 S368 phosphorylation by PKC isoforms

As an integral gap junction protein, Cx43 plays an important role in intercellular communication, providing a path of least resistance for small molecules and secondary messengers to transit between adjacent cells (2). The phosphorylation/dephosphorylation of Cx43 in its soluble C-terminal tail is a critical regulator of intercellular communication (90). For example, the inositol 1,4,5-trisphosphate receptor (IP_3_R) interacts with, and regulates the phosphorylation of Serine-279/282 in Cx43 in mouse ventricular cardiomyocytes. IP_3_R is a large conductance cationic channel that plays an essential role in controlling Ca^2+^ exchange. Blocking IP_3_R with antagonists suppresses Cx43 phosphorylation at S279/282 and hinders intercellular communication (91). As described above, various kinases have been implicated in Cx43-mediated intercellular communication such as the mitogen-activated protein kinase (MAPK), Src, and the Protein Kinase C isozymes (92).

Protein kinase Cs, or PKCs, are a superfamily of Serine/threonine kinases that partake in various signaling transduction pathways and cellular functions such as cell proliferation, migration, and apoptosis (93–95). The PKC family consists of structurally homologous isozymes which are divided into three groups based on their secondary messenger requirements namely: classical, novel, and atypical (96). Classical PKCs, including isoforms α, β_I_, β_II_, and γ, require Ca^2+^ and a phospholipid such as DAG for activation in which they harbor a C2 domain that binds Ca^2+^ and a C1 domain that binds DAG (97). Novel PKC isoforms include δ, ε, η, θ, and μ which also contain a C1 domain and a C2-like domain but only require DAG for activation. Atypical PKCs, including ζ, ι, and λ, lack a Ca^2+^ binding domain and have a C1-like domain that is incapable of binding to DAGs but are activated by other lipid mediators such as sphingosine 1-phosphate (98).

There are multiple downstream targets of PKCs which in turn activate signaling pathways that regulate various cellular activities including cell migration, invasion, survival, and proliferation. PKCs typically recognize 5 consensus motifs: (R/K)X(*S*/*T*); (R/K)(R/K)X(*S*/*T*); (R/K)XX(*S*/*T*); (R/K)X(*S*/*T*)R/K; (R/K)XX(*S*/*T*)R/K; or (*S*/*T*)XR/K (99). For example: EGF receptors are recognized by PKCα through a RKAT sequence corresponding to the motif (R/K)(R/K)X(*S*/*T*) while PKC δ recognizes a RILT sequence on the insulin receptor tyrosine kinase corresponding to the consensus recognition motif (R/K)XX(*S*/*T*) (100). A well-established signaling pathway that is stimulated by PKC is the Raf/Mek/Erk cascade through which survival and proliferation is regulated in cancer cells (101). PKC overexpression can induce migration and invasion in intestinal epithelial cells while repressing apoptosis through the Ras/PKCι/Rac1/MAPK kinase signaling pathway (102). In cardiomyocytes, the JAK/Stat pathway is activated in response to mechanical stretch, and it has been observed that PKC gets activated by mechanical stretch and subsequently phosphorylates Stat1 and Stat3 (103). Similarly, PKCs are involved in the downregulation of p38 MAPK signaling, stabilization of steroid receptor co-activator (src-3), and inhibition of Akt activation in response to growth factors (104–106). These findings highlight the plethora of signaling pathways that PKCs regulate making them essential regulators of cellular function.

Cx43 is directly phosphorylated by PKCs at S368 (107). This phosphorylation leads to a reduction in gap junction permeability affecting intercellular communication (107). The C-terminal tail of Cx43 contains RXSSR repeats that are recognized as PKC phosphorylation consensus sites (108). *In vitro* phosphorylation assays with PKC followed by peptide sequencing demonstrated that S368 of Cx43 is phosphorylated by PKC after treatment with 12-*O*-tetradecanoylphorbol-13-acetate (TPA) (107), a small diacylglycerol mimetic that induces PKC activity (109, 110). Ectopically expressing wild-type Cx43 in Cx43 deleted cells followed by TPA treatment decreases single channel permeability while expressing a Cx43 S368A mutant version does not affect channel permeability, demonstrating an integral role for Cx43 S368 phosphorylation by PKC to regulate channel permeability and intercellular communication (107).

Further studies observed that S368 phosphorylation reduces Cx43 hemichannel pore diameter/cross sectional area to reduce the incidence of the 100 pS conductance state and an increase in the 50 pS conductance state following TPA treatment. Interestingly, consistent with this notion, mutation of S368 to alanine reduces the incidences of 55–70 pS channels and also reduces the selective permeability of the gap junctions for a cationic dye NBD-M-TMA (111). Similarly, other strategies to reduce S368 phosphorylation such as inhibition of PKC or expression of the C-terminal tail fragment reduces the 50–70 pS conductance state (111). Together, these observations suggest a critical role for S368 phosphorylation in maintaining channel conductance and selective permeability. Consistent with this notion, if all six Cx43 subunits within a hemichannel are phosphorylated at S368, they are shown to lose permeability to sucrose which has a molecular weight of 342 daltons. Whereas, a smaller compound, ethylene glycol with a molecular weight of 62 daltons, was still permeant through these fully phosphorylated hemichannels (112). However, selective permeability of Cx43 hemichannels seems to be independent of the molecular weight of the permeant molecule. When Cx43 was expressed in *Xenopus* oocytes, hemichannels showed higher uptake of ethidium (310 daltons) but restricted uptake of smaller compounds such as glutamate (147 daltons) and Urea (60 daltons) (113). Hence, the selective permeability of Cx43 hemichannels, and its regulation through S368 phosphorylation, to a particular molecule may need to be individually characterized as molecular weight may not be the only determinant involved in dictating selectivity. At the structural level, phosphorylation of Cx43 by PKC at S368 promotes a conformational change in the C-terminal tail of Cx43, while a S368A substitution blocked this confirmational change mediated by PKC (114). Studies using a scrape loading dye transfer assay (SL/DT assay) in Cx43 deleted fibroblasts ectopically expressing wild-type Cx43 revealed that dye coupling was reduced in cells reconstituted with Cx43 while dye coupling was not reduced when cells were reconstituted with a Cx43 S368A mutant suggesting that PKC-mediated S368 phosphorylation of Cx43 is a pivotal player in intercellular communication. In further support of this notion, intercellular communication increases in the presence of PKC inhibitors such as Go6976 (107). Furthermore, TPA-induced downregulation of intercellular communication is reversible. Once TPA activates classical and novel PKC isoforms, these isoforms including PKCε and PKCδ translocate from the cytosol to the plasma membrane where Cx43 is embedded and phosphorylates S368 (115, 116). However, prolonged TPA activation can lead to the degradation of the PKC isoforms α, δ, ε, and ι through the ubiquitin-proteasome system upon which intercellular communication recovers (117, 118). In the rat liver epithelial cell line IAR20, inhibition of the proteasomal degradation pathway with MG132 prolongs PKC activity and prevents intercellular communication. However, inhibiting both the proteasomal degradation pathway and PKCs, using a non-specific PKC inhibitor GF109203X, reverses the effect of MG132 (119). These studies provide support that PKC regulates intercellular communication through phosphorylating Cx43 on S368.

Several studies have been conducted that reveal regulation of Cx43 phosphorylation at S368 by multiple PKC isozymes. For instance, PKCδ, a novel PKC isoform, has been shown to phosphorylate S368 leading to gap junction internalization and degradation (120). Using fluorescence energy transfer (FRET) to study the spatiotemporal localization of Cx43 following S368 phosphorylation by PKCδ, treatment with a phorbol ester compound PDBu led to a decrease in FRET signal within the gap junction followed by internalization of S368 phosphorylated Cx43 containing vesicles that colocalized with proteasomal and lysosomal vesicles suggesting that Cx43 S368 phosphorylation by PKCδ leads to its internalization and degradation through the proteasomal and lysosomal degradation pathway (120). PKCδ was shown to directly interact with Cx43 in response to fibroblast growth factor 2 (FGF-2) treatment in osteoblast cell lines (121). FGF-2 treatment induces phosphorylation of PKCδ at Threonine-505, binding of the C-terminal tail of Cx43 by PKCδ, and phosphorylation of Cx43 S368. Inhibition of PKCδ with Rottlerin abolished FGF-2 induced Cx43 phosphorylation, demonstrating that PKCδ regulates Cx43 phosphorylation in response to FGF-2 (121).

Another novel PKC isoform that has been linked with FGF-2-mediated Cx43 phosphorylation is PKCε. Upon treatment of cardiomyocytes with FGF-2 and Phorbol 12-myristate 13-acetate (PMA), another DAG mimetic, Cx43 and PKCε colocalized at sites of intercellular connection. Subsequent coimmunoprecipitation studies revealed that PKCε physically interacts with Cx43 and an increase in C-terminal tail phosphorylation (using an antibody that recognizes multiple phosphorylated residues) was observed but direct regulation of Cx43 S368 phosphorylation could not be determined (122). In a subsequent study, cardiomyocytes ectopically expressing a dominant-negative version of PKCε reduced Cx43 phosphorylation levels in response to FGF-2 treatment as observed by incorporation of radiolabeled ATP. FGF-2 treatment was also shown to decrease gap junctional coupling as assessed by monitoring fluorescent dye (6-CF) transfer between cardiomyocytes (123). Ischemic preconditioning (IPC) in the heart is known to induce cardioprotection through gap junction coupling (56, 124). During IPC, the heart is subjected to brief periods of ischemia to increase its resistance toward sustained ischemia (125), and is one of the most effective methods to protect against myocardial ischemic injury (126). Isolated rat ventricular tissues that underwent ischemic preconditioning show enhanced Cx43:PKCε complex formation as well as PKCε-mediated phosphorylation of Cx43 at S368 (127). FGF-2 confers cardioprotective effects to ischemic injury in a PKC-dependent manner. Rat hearts perfused with FGF-2 *ex vivo* show upregulated levels of phosphorylated Cx43 on S368 in intercalated discs suggesting a link between phosphorylation of Cx43 at PKC target sites such as S368 and FGF-2 induced cardioprotection against ischemia (128). PKC can also be activated by cholesterol, and cholesterol treatment reduces dye transfer in the H9c2 cardiomyocyte cell line while PKC inhibition partially restores dye transfer capacity in these cells. Cholesterol treatment upregulates Cx43 S368 phosphorylation in a PKC-dependent manner as PKC antagonists reduces cholesterol induced Cx43 S368 phosphorylation (129). Similarly, lysophosphatidylcholine (LPC) treatment in H9c2 cells induces PKCε dependent activation of Cx43 S368 phosphorylation and the loss of gap junction intercellular communication through enhancement of Cx43 ubiquitination and proteasomal degradation. Furthermore, treatment with a PKCε specific inhibitor, eV1-2, prevents the LPC induced reduction of intercellular communication (130).

In addition to gap junctional Cx43, hemichannels containing Cx43 can also be phosphorylated at Serine 368 by PKCs (131). Open hemichannels can conduct ion movement including Na^+^, Ca^2+^, and K^+^ (132). Hemichannel opening can be activated by various stimuli such as metabolic inhibition, positive membrane potential, phosphorylation at S368 by PKC, and stimulation by ryanodine (Ryr) receptors (133–135). In ventricular cardiomyocytes, influx of Ca^2+^ combined with the activation of Ryr receptors leads to hemichannels opening. Ryr receptors interact with Cx43 hemichannels through its C terminal tail (134). One interesting link between PKC and Cx43 hemichannels is found in mitochondria where Cx43 translocates to during stress conditions such as hypoxia (136). Under hypoxic conditions in cardiomyocytes, PKCε mediates interaction between mitochondrial Cx43 hemichannels and the ATP regulated mitochondrial potassium ion channel Kir6.1. Phosphorylation of mitochondrial hemichannels increases in response to hypoxia and phosphorylation of Cx43 S262 is involved in the interaction between Cx43 and Kir6.1 in H9c2 rat heart cells (136). Since PKCε phosphorylates both S262 and S368, it is interesting to speculate that the mitochondrial hemichannels may also be phosphorylated at S368 and may influence the interaction between Cx43 and Kir6.1. In studies assessing the protection conferred by FGF-2 against ischemic insult, PKCε translocation was reported to increase in the subsarcolemmal mitochondria where Cx43 S262 and S368 phosphorylation is increased by 30- and 8-fold, respectively. PMA stimulation also increased Cx43 hemichannel phosphorylation which is reversed by the PKCε inhibiting peptide εV1-2 (131). In addition, mitochondrial hemichannel opening is reduced in cardiomyocytes isolated from mice harboring a knockin mutation for Cx43 S368A suggesting further that mitochondrial hemichannels are targeted by PKC in cardiac cells (137), however, more research is necessary to fully understand the role of Cx43 composed hemichannels in regulating mitochondrial biology.

PKCγ is a classical PKC isoform and is most abundantly expressed in neurons, the retina, and the eye lens (138, 139). As a classical PKC, it is activated by DAGs (140). Interestingly, PKCγ can also be activated by oxidative stress, such as exposure of cells to H_2_O_2_, which stimulates the C1 domain of PKCγ to translocate to the plasma membrane where PKCγ targets Cx43 for phosphorylation on S368. PKCγ physically interacts with Cx43 and drives the disassembly of Cx43 from the membrane leading to a reduction in intercellular communication (141). Interestingly, PKCγ led disassembly of Cx43 requires zonula occludens protein-1 (ZO-1), a tight junction protein. In the presence of TPA, ZO-1 interacts with Cx43 through its PDZ binding domain which lies in close proximity to S368 on Cx43. PKCγ is able to interact with Cx43 even in the absence of ZO-1 or TPA stimulation. Although Cx43 that is complexed with PKCγ in ZO-1 depleted cells lacks S368 phosphorylation, suggesting that ZO-1 may be required for PKCγ to phosphorylate Cx43 on S368 (142).

**FIGURE 2:**
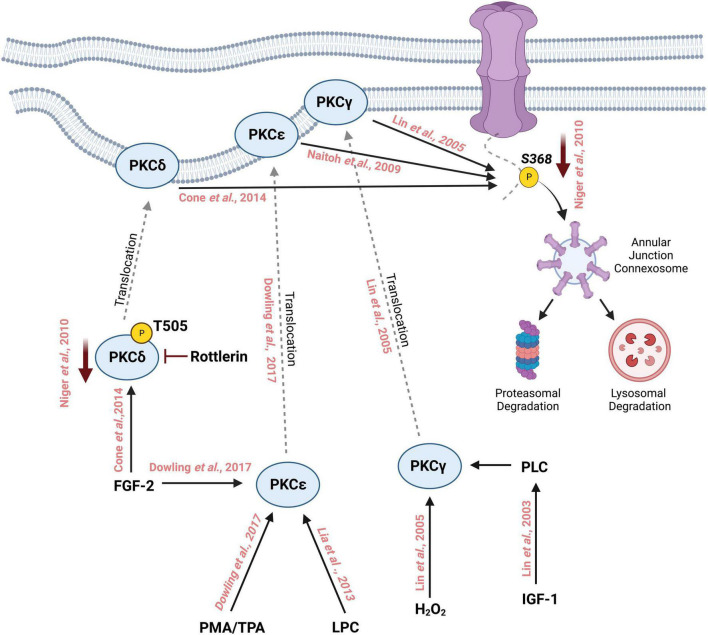
Schematic representation of phosphorylation of Cx43 by different isoforms of Protein Kinase C on Serine 368 followed by its degradation through the lysosomal and proteasomal degradation machinery. Treatment with phorbol myristate acetate (PMA) or 12-*O*-tetradecanoylphorbol-13-acetate (TPA) activates PKCε which then translocates to the cell membrane from the cytosol. Similarly, fibroblast growth factor-2 (FGF-2) and lysophosphatidylcholine (LPC) can also activate PKCε which in turn interacts with the C-terminal tail of Cx43 and phosphorylates it at S368. FGF-2 can activate and increase the phosphorylation of PKCδ at T505, which then translocates to the cell membrane where it phosphorylates Cx43 at S368. Inhibition with rottlerin reduces the activity of PKCδ which in turn reduces the phosphorylation of S368. PKCγ is activated by oxidative stress such as the addition of H_2_0_2_ thereby inducing the translocation of PKCγ to the cell membrane where it interacts with Cx43 and phosphorylates S368. Insulin-like growth factor 1 (IGF-1) activates PKCγ leading to the phosphorylation of Cx43 S368.

In addition, growth factors such as insulin-like growth factor (IGF-I) induce phosphorylation of Cx43 mediated by PKCγ. IGF-1 triggers the production of DAGs through Phospholipases (PLCs) which activate the classical PKCs including PKCγ (143). PKCβ, another classical PKC, has been examined for its role in modulating gap junction intercellular communication through Cx43. Rat R6 fibroblasts overexpressing PKCβI did not affect TPA-induced suppression of intercellular communication, suggesting that PKCβ may not play a major role in Cx43-mediated intercellular communication. Further experiments using the PKCβ-specific inhibitor LY379196 showed no change in intercellular communication (144). Similarly, PKCμ was shown to be of minor importance in TPA-mediated downregulation of intercellular communication in this study, since repeated TPA treatments showed little downregulation of PKCμ unlike other classical isozymes such as PKCα (144). Based on these studies, it can be concluded that several isoforms of PKC play a major role in regulating intercellular communication through the phosphorylation of Cx43 S368 (Figure 2). Further characterization of the roles for various PKC isoforms in regulating Cx43 phosphorylation and subsequent intercellular communication is necessary to understand the complex signaling pathways controlling gap junction function and intercellular communication. The relationship between PKC isozyme expression and Cx43 S368 phosphorylation should be further explored in various organs since the abundance of PKC isozymes differs between tissues and organ. For instance, the expression of PKC α and β is the highest in the human brain while PKCδ is abundantly expressed in the adrenal gland (145).

## Cx43-mediated intercellular communication and cardiac conduction

Action potential propagation through both cardiomyocytes and non-cardiomyocytes in the ventricles has long been thought to be mediated by Cx43 (146). Genetic studies have shown that cardiac-specific Cx43 knockout produces ventricular tachycardia, prolonged PR interval, slowed cardiac conduction, and sudden cardiac death (147, 148). Cx43 is found in the intercalated discs of ventricular cardiomyocytes and is important for electrical impulse propagation. Translocation of Cx43 to and from the plasma membrane is tightly regulated and it has a high turnover rate (25). Any changes to Cx43 distribution can affect intercellular communication and lead to lethal arrhythmias such as ventricular tachyarrhythmia (149). Since phosphorylation of Cx43 at S368 can regulate gap junction pore opening, translocation, and degradation, it suggests that Cx43 phosphorylation at this residue is essential for proper cardiac conduction. Studies in guinea pig hearts following induction with calcium revealed an increase in Cx43 S368 phosphorylation and decrease in gap junction electrical conductance as well as arrhythmias and slower action potential conductance, indicating that Cx43 S368 phosphorylation and cardiac conduction were intimately linked (150). In addition, other phosphorylation sites within the C-terminal tail of Cx43, such as 325/328/330, are also important for gap junctional conductance and the prevention of arrhythmia. Germline knock-in mouse models with phosphomimetic mutation of these three sites display a reduction in arrhythmia and are resistant to gap junctional remodeling (151). Based on these studies, it became widely accepted that Cx43 is indispensable for cardiac conduction.

However, more recent studies have emerged that question this view. For instance, germline Cx43 knockout mice die at birth due to asphyxia as a result of a defect in the pulmonary outlet and not due to arrhythmia (152). Furthermore, cardiac-specific knockout mice survive up until the fourth week of postnatal life and ultimately die due to fatal arrhythmias (148). However, these mice appear to retain a normal heart rhythm. In humans, Cx43 mutation is tied to Oculodentodigital dysplasia which is a rare congenital autosomal disorder characterized by phenotypic variability. ODDD linked Cx43 mutations G21R and G138R colocalize and coimmunoprecipitate with wild-type Cx43 (153). However, these mutants display a dominant negative effect on gap junctional conductance and yet individuals with these mutations maintain normal cardiac conduction. Similarly, a knockin mouse model where S368 is substituted with an alanine and thus cannot be phosphorylated does not show significant changes in terms of gap junction localization and Cx43 abundance. These studies suggest that Cx43 might not be essential to action potential propagation between cardiomyocytes.

A new model of action potential propagation involving gap junctional structure termed “ephaptic coupling” has emerged which suggests that ionic currents are transmitted between cardiomyocytes through the confined extracellular space at the perinexus which lies at the edge of the gap junction plaque in which adjacent cells are not connected (154–156). Such coupling does not require a continuous pore or direct contact between cells mediated by gap junctions. Manipulation of extracellular volume in the hearts of guinea pigs showed that an increase in extracellular space reduces action potential velocity providing further precedence to determine if other modes of intercellular coupling/communication are present that do not involve the conventional electrotonic mode of conduction through gap junctions but rather through ephaptic coupling (157). Super resolution microscopy of rat ventricular myocytes has shown that the perinexus consists of clusters of Cx43 hemichannels and Na_v_1.5 sodium channels (157). Na_v_1.5 sodium channels contain a β1 subunit which is non-pore forming but acts as an adhesion molecule between cells (158), and also localizes with Cx43 hemichannel clusters within the perinexus. Inhibition of the β1 subunit results in loss of adherence at the perinexus cleft and an increase in the perinexus space in guinea pig ventricles. This de-adherence reduces sodium ion currents in gap junctions adjacent to sodium channels but does not affect the whole cell sodium current further supporting the ephaptic coupling hypothesis. Inhibition of the β1 subunit in guinea pig hearts slows cardiac conduction and causes arrhythmia including prolongation of the QT interval (159). Mathematical modeling suggests that membrane spacing of <30 nm is required for ephaptic coupling (156). β1 subunit adhesion is predicted to provide membrane spacing of less than the 30 nm limit enabling ephaptic coupling to occur (159). Gap junctional structure can therefore support cardiac conduction by providing a perinexus region in which sodium ion channels can propagate action potential through ephaptic coupling. Interestingly, expression level of Na_v_1.5 is reduced in hearts of mice heterozygous for Cx43 (160) and at the cellular level, this reduction in Na_v_1.5 was observed in regions devoid of Cx43. These mice also had slowed and dispersed conduction suggesting the importance of Cx43 in the expression and function of sodium ion channels in the heart (160). Hence, both electrotonic coupling and ephaptic coupling through Cx43 could exist as a mixed mode of action potential transmission and the balance between these two mechanisms could be key in clarifying our understanding of the role of Cx43 in cardiac conduction (161).

## PKC and Cx43 expression in the healthy heart

The heart predominantly expresses three connexin isoforms, namely Cx40, Cx43, and Cx45, of which Cx43 is the most abundantly expressed (162). Cx43 expression initiates at E8.5 in the mouse embryonic heart and gradually increases throughout development and is found in both the adult atria and ventricles (163). Cx43 is specifically expressed in the trabeculations of embryonic ventricles starting at E10.5 and is expressed by the entire myocardium in the adult heart (42, 163). Similar to its expression pattern, phosphorylation of Cx43 at S368 increases during embryonic development in the heart and it increases significantly in the adult heart. Studies assessing Cx43 S368 phosphorylation between E14.5 embryos and 12-month-old mice revealed an increase in pS368 levels in the adult hearts while the relationship between embryonic and adult tissue was reversed in other tissues such as skin and cornea (164). This increment in phosphorylation of Cx43 in embryonic and adult hearts may be explained by the observed increase in expression of PKC isoforms that directly phosphorylate Cx43 at S368. For example, PKCε expression increases throughout mouse embryonic development in the heart (165). Furthermore, human heart tissues express several PKC isoforms. Western blot and immunohistochemical analysis show that classical isoforms PKCα and PKCβ are present in the human heart while PKCγ is absent. Novel PKC isoforms δ, ε, η, and the atypical PKC isoform PKCμ are also present in cardiomyocyte homogenates (166). Therefore, co-expression of PKCs and Cx43 are consistent with a regulatory network between PKCs and Cx43 S368 phosphorylation.

## PKC-Cx43 S368 in the diseased heart

### Ischemia

Myocardial ischemia, which refers to the reduction of blood flow and oxygen to the heart, can occur following coronary artery blockage due to the accumulation of atherosclerotic plaques (167). It is estimated that myocardial ischemia affects around 126 million individuals globally (168). Ischemic insult reduces gap junction intercellular communication between cardiomyocytes which affects electrical impulse propagation leading to arrhythmias (29). Cx43 degradation is dependent upon AMP activated protein kinase (AMPK) during initial periods of ischemia while the later periods require the autophagy regulating protein Beclin-1. Furthermore, degradation of Cx43 due to ischemia also led to an impairment of gap junction intercellular communication in HL-1 mouse cardiomyocytes. Inhibiting autophagy restored intercellular communication indicating that autophagy is involved in ischemia induced Cx43 degradation (29). Reduction of gap junction intercellular communication post ischemia is also observed in neonatal rat heart myofibroblasts. Interestingly, prolonged ischemia led to the opening of hemichannels while gap junctions remained closed. When Cx43 hemichannels are inhibited with Gap26, a connexin derived peptide, infarct size is significantly reduced in isolated perfused rat hearts which suggests that open hemichannels could contribute to the propagation of infarct promoting signals across the heart (169). Studies performed in rat models of ischemia perfusion found an increase in Cx43 S368 phosphorylation at the intercalated discs (ID) of cardiomyocytes which triggers the ubiquitination and subsequent degradation of Cx43 (170). This reduction in Cx43 at the IDs contributes to the electrical uncoupling and reduction in conduction velocity, a hallmark of ischemic hearts (171). In another study in which wild-type mice underwent no-flow ischemia also showed a significant increase in cardiac Cx43 S368 phosphorylation as compared to control mice while total Cx43 levels decreased. This degradation of Cx43 was promoted by 14-3-3 theta which is involved in anterograde transport of Cx43 (70). 14-3-3 theta binds to Cx43 when phosphorylated at S373 (172). Interestingly, S373 acts as a gatekeeper to S368 phosphorylation and S373A substitution impairs PKC induced phosphorylation of S368 in cells exposed to ischemic conditions (173). In contrast to these studies, other studies suggest ischemia has the opposite effect on gap junction intercellular communication. For instance rats subjected to left anterior descending coronary artery (LAD) occlusion showed higher Cx43 levels in the intercalated discs and dye transfer assay revealed an increase in gap junction-mediated intercellular communication after 30 min of LAD occlusion (174). Similarly, adult rat hearts exposed to ischemia undergo electrical uncoupling during which reduced Cx43 phosphorylation is observed. Reperfusion, on the other hand, increases phosphorylated Cx43 at the intercalated discs (25). Mass spectrometry studies on rat hearts that underwent ischemia show that S368 undergoes dephosphorylation within 15–30 min following ischemia, corresponding to the time interval that the majority of gap junction uncoupling occurs. Treatment with an antiarrhythmic peptide analog rotigaptide (ZP123) suppresses dephosphorylation of S368 after 30 min of ischemia and prevents gap junction uncoupling (175). One mechanism that may promote an increase in Cx43 phosphorylation following reperfusion is through PKCs, such as PKCα and PKCε, that translocate to the cell membrane upon myocardial ischemia reperfusion. PMA treatment of rat hearts promotes the translocation of PKC isozymes, including α, δ, and ε, to the cell membrane (176). All three of these isozymes have been implicated in Cx43 S368 phosphorylation at the cell membrane. In cryoinjured left ventricular tissue treated with αCT-1, a peptide containing nine amino acids of the Cx43 C-terminal tail, an acute increase in Cx43 S368 phosphorylation is observed (177). However, control hearts treated with αCT-1 do not show any change in S368 phosphorylation suggesting that Cx43 S368 phosphorylation is dependent on injury (177, 178). αCT-1 enhances PKCε-mediated phosphorylation of Cx43 S368 in the injured hearts and prevents arrhythmia (178). In response to injury, PKCε translocates and phosphorylates Cx43 S368. Interestingly, ischemic injury also induces translocation of PKCε in the heart of conscious rabbits (179). Similarly, another study demonstrated that ischemic preconditioning in adult rat hearts promoted PKCε-mediated Cx43 S368 phosphorylation and suppresses Cx43 lateralization (180). Overall, these findings suggest that the increased translocation of PKCs to IDs during ischemia contributes to electrical uncoupling through enhanced Cx43 S368 phosphorylation.

### Myocarditis

Myocarditis is defined as inflammation of the heart muscle which can reduce its ability to pump blood (181). Global trends indicate that myocarditis affected over 3 million people in 2017 alone (182). Cx43 S368 phosphorylation is upregulated in acute myocarditis, which accounts for a majority of sudden cardiac deaths in people without any prior heart conditions (183, 184). Myocarditis is accompanied by abnormal ECG patterns the most common of which is sinus tachycardia associated with non-specific ST/T-wave variations. A study of ECG patterns found in myocarditis revealed multiple characteristics such as depression in precordial and limb leads in the PR segment, pericarditis pattern in the ST segment, and a prolonged QT interval (185). Utilizing a model of rat experimental autoimmune myocarditis (EAM), Cx43 S368 phosphorylation was shown to be elevated in this model (184). Downregulation of Cx43 S368 phosphorylation improves gap junction intercellular communication and reduces the prolonged QRS interval. Since the EAM model is a T cell-mediated inflammatory disorder of cardiac tissues, this study further perfused isolated rat hearts with inflammatory cytokine IL-1β and found that perfusion with IL-1β induces Cx43 S368 phosphorylation in normal rat hearts. EAM models also show upregulation of PKCα in the rat heart, which may provide a mechanism for the increased Cx43 S368 phosphorylation levels. Consistent with this notion, intraperitoneal administration of the PKC inhibitor Ro-32-0432 reduces pro-inflammatory cytokines such as IL-1β which activates Cx43 S368 phosphorylation, as well as reduces biomarkers of heart failure in the EAM model (186). These observations further corroborate the relationship between PKC isozymes and Cx43 S368 phosphorylation in the context of inflammatory cytokines and myocarditis. However, further studies are required to assess the significance of this relationship in myocarditis and if the PKC/Cx43 pathway can serve as a novel therapeutic target to alleviate consequences associated with myocarditis.

### Cardiomyopathy

Cardiomyopathy encompasses a variety of heart conditions that influence the ability of the heart to pump blood into systemic and pulmonary circulation, the most common of which is hypertrophic cardiomyopathy. This condition is caused by the thickening of the left ventricle chamber wall (187, 188). PKC isozymes have been extensively studied in relation to hypertrophy. For instance, adult mice overexpressing classical PKCβ exhibit mild and progressive ventricular hypertrophy, while overexpression in neonates leads to sudden cardiac death (189). Similarly, neonatal cultured rat cardiomyocytes overexpressing PKCα show hypertrophic cardiomyocyte growth as well as induction of the hypertrophy marker atrial natriuretic factor, whereas overexpression of PKCδ and PKCε does not lead to cardiomyocyte hypertrophy. Furthermore, a kinase-inactive mutant of PKCα did not affect cardiomyocyte hypertrophy, indicating downstream phosphorylation events mediated by PKCα are required for its role in cardiac hypertrophy (190). Heart explants from dilated cardiomyopathy and ischemic cardiomyopathy patients have increased levels of PKCα and PKCβ. Immunoblotting experiments revealed a greater than 40% increase in PKCβ levels at the membrane, PKCα levels increase by 70% in the cardiomyopathic left ventricles, while PKCε expression does not significantly change (191).

The DAGs such as PMA have been used to induce hypertrophy since PMA activates most PKCs. Neonatal rat ventricular cardiomyocytes (nrCMCs) treated with PMA show a significant increase in cell size. These cardiomyocytes also have increased markers of pathological hypertrophy such as Nppa, Acta1, and Serca2a. NrCMCs treated with PMA display tachyarrhythmia which is also a characteristic of hypertrophy (192). Given that PKCs activated by PMA directly phosphorylate Cx43 at S368 as described above, one might anticipate that hyperphosphorylation of S368 on Cx43 would promote hypertrophy. A rat model of pressure-overload hypertrophy revealed an initial increase in Cx43 S368 phosphorylation which was followed by a decline after 8 weeks, and these animals were prone to sustained ventricular tachycardia (193). In contrast, it has also been reported that rats treated with monocrotaline to induce right ventricular hypertrophy show an increase in non-phosphorylated Cx43 and cytosolic annular gap junctions (194). Using inhibitors against protein phosphatase 1 (PP1) suppresses cardiac hypertrophy and heart failure (195). Consistent with this observation, treatment with PP1 inhibitors such as Okadaic acid prevents ischemia-induced Cx43 dephosphorylation in cardiomyocytes (196). Based on these findings, PKC activity may need to be carefully modulated in the heart to maintain Cx43 S368 phosphorylation in equilibrium to ensure appropriate gap junctional communication levels. A more apparent effect of the PKC/Cx43 relationship in cardiomyopathy is observed in mice treated with Furazolidone for 30 weeks to induce dilated cardiomyopathy (197). Mitochondrial dysfunction is a major contributor to dilated cardiomyopathy (198). Furazolidone treatment reduces myocardial mitochondrial Cx43 S368 phosphorylation along with PKCε levels leading to mitochondrial dysfunction. Treatment with PMA, a PKC activator, increases PKCε activity and partially reverses Furazolidone inhibition of Cx43 S368 phosphorylation. In contrast, treatment with the Cx43 inhibitor, 18β-glycyrrhetinic acid, inhibits the effect of PKC on mitochondrial dysfunction indicating that PKC-mediated Cx43 S368 phosphorylation is essential to reduce mitochondrial dysfunction in dilated cardiomyopathy and therefore could serve as a novel therapeutic target for cardiomyopathy (197).

### Arrhythmias and heart failure

Arrhythmias such as atrial and ventricular tachycardia can lead to heart failure in which the heart fails to pump sufficient blood to the systemic and pulmonary circulation. Heart failure affects around 26 million people worldwide and is considered a global pandemic (199). About 30% of heart failure patients suffer from atrial fibrillation while 50% of sudden death attributed to heart failure is accompanied by ventricular arrhythmias (200–202). Many studies have examined the expression and phosphorylation status of Cx43 in atrial fibrillation and congestive heart failure in various species. However, there are significant variations between species. For instance, expression of Cx43 during atrial fibrillation decreases in rabbits while it increases in dogs (203, 204). Interestingly, congestive heart failure in dogs reduces the phosphorylation of Cx43 at S368 and overall Cx43 in the atria. Ventricular Cx43 undergoes dephosphorylation and lateralization, inducing arrhythmia and slowing conduction during heart failure (205, 206). Along with changes in expression and phosphorylation levels of Cx43, several cardiac diseases such as arrhythmogenic right ventricular cardiomyopathy (ARVC) are accompanied by remodeling of Cx43 (207). Heart failure patients have reduced levels of Cx43 in the gap junction plaques found at the intercalated discs and an increase in the number of Cx43 in the lateral walls of cardiomyocytes (208, 209). Lateralization of Cx43 is in contrast with the normal distribution of Cx43 which is concentrated at the intercalated disc in the ventricular myocardium (210). Cx43 hemichannels require positive membrane potential of >+50 mV to open. During systole, cardiomyocytes have an elevated level of cytoplasmic Ca^2+^ which lowers the membrane potential required to open hemichannels to +30 mV (211). Entry of Ca^2+^ into cardiomyocytes further induces the sarcoplasmic reticulum to release more Ca^2+^ into the cytoplasm that can lead to the activation of hemichannels. A single open hemichannel can display high conductance of around 220 pS which can allow for the movement of ions such as Na^+^ and K^+^ (132). During pathological conditions, the sarcoplasmic reticulum is spontaneously triggered to release Ca^2+^ which leads to the opening of hemichannels and delayed afterdepolarizations that further promotes arrhythmia (212, 213). Caffeine can induce sarcoplasmic reticulum release of Ca^2+^ through the activation of ryanodine receptor channels (Ryr2) (214). Ryr2 interacts with and regulates Cx43 hemichannel opening due to elevated Ca^2+^ levels in cardiomyocytes. Ventricular cardiomyocytes isolated from heart failure patients display spontaneous Ca^2+^ release and hemichannel opening along with delayed afterdepolarizations. In addition, adrenergic stimulation of arterially perfused tissue wedges had higher occurrences of delayed afterdepolarizations which were suppressed by Gap19, a Cx43 hemichannel specific inhibitor (134). This indicates that Cx43 hemichannel lateralization and opening contributes to arrhythmogenic activities. Significant levels of Cx43 lateralization is also observed in mouse models of Duchenne muscular dystrophy (DMD) which is characterized by the loss of dystrophin that stabilizes the sarcolemma in myocytes (207). Cardiac arrhythmias are often seen in young and adolescent individuals with DMD (215). Cx43 lateralization is also observed in mouse models of DMD. Upon isoproterenol treatment, DMD mice develop arrhythmias and die within 24 h. However, treatment with the hemichannel inhibitors Gap19 or Gap26 protects DMD mice from arrhythmogenesis and death suggesting that Cx43 hemichannel activity and lateralization are linked with arrhythmia *in vivo* (207).

Disruption of Ca^2+^ homeostasis and Cx43 hemichannel function are also tied to ARVC that is associated with mutations in desmosome proteins such as desmoglein-2, desmoplakin, and Plakophilin-2 (PKP2) (216). ARVC or arrhythmogenic right ventricular dysplasia (ARVD) is characterized by ventricular tachycardia and sudden cardiac death (217). PKP2 knockout mice display higher levels of Ca^2+^ levels in the cytoplasm and sarcoplasmic reticulum along with increased duration of Ca^2+^ transient currents in right ventricular myocytes (216). Susceptibility to arrhythmia is also significantly higher in PKP2 knockout mice. Interestingly, hemichannel specific inhibition with Gap19 normalizes Ca^2+^ homeostasis. Cx43 reduction also decreases Ca^2+^ permeability and cytoplasmic Ca^2+^ accumulation in right ventricles of PKP2 knockout mice, which was also observed following treatment with the PKC inhibitor GF 109203X (216). Single-molecule localization microscopy indicated that PKC clusters were reduced in the intercalated disc of the PKP2 knockout hearts. Cx43 S368 phosphorylation in the PKP2 knockout group was also lower in both the right and left ventricle which could be due to a reduction in PKC levels at the intercalated disc since PKC phosphorylates Cx43 at S368 (216). Total Cx43 was higher in the PKP2 knockout group as compared to the control group. It is interesting to speculate if this may be due to a reduction in Cx43 hemichannel degradation as S368 phosphorylation is known to play a role in Cx43 degradation (218). Utilizing scanning electron microscopy and Fluoro-Gold labeled Cx43, Cx43 hemichannel abundance was found to be higher in PKP2 deficient hearts (219). However, these Cx43 hemichannels are present in the intercalated disc as “orphan Cx43” and are not docked to form a gap junction. In addition, a widening of the intercellular space in the gap junction plaque in PKP2 deficient hearts suggests a loss of gap junction connection in these spaces (219). Loss of gap junctions in the intercalated discs is also seen in Naxos disease which is a recessive form of ARVC (220). Similarly, Cx43 expression is reduced in cardiomyocytes of cardiomyocyte-specific desmoplakin deficient mice which is compounded by a significant reduction of Cx43 S368 phosphorylation and function (221). Another intriguing observation in patients with ARVC is the reduction in Na_v_1.5 sodium channels which is predicted to mediate ephaptic coupling in the hemichannels in the perinexus (222, 223). Therefore, Cx43 function, mediated by both expression levels and S368 phosphorylation is a critical regulator of Ca^2+^ homeostasis in the heart.

The PKC-mediated hyperphosphorylation of Cx43 at S368 in isolated normal heart tissue can also induce lateralization of Cx43 from the intercalated disc to the lateral membrane of the ventricular myocardium (224). This hyperphosphorylation is also associated with arrhythmia with a prolonged QRS complex (184). Immunohistochemical analysis showed redistribution of Cx43 to the lateral membrane in hearts with hyperactivated PKC in response to PMA treatment. Suppression of PKC activity with the PKC inhibitor Ro-32-0432 significantly decreased phosphorylation of Cx43 S368 and suppressed this lateralization (224). In addition, Cx43 lateralization in left ventricle occurs following isoproterenol treatment (225). Isoproterenol is a sympatho-mimetic agent capable of inducing atrial fibrillation and heart failure. Taken together, these studies indicate an important relationship between PKC and Cx43 S368 phosphorylation in arrhythmogenesis promoted by hemichannel lateralization in the heart. Several other PKC isozymes are upregulated in failing hearts including PKCα and PKCε as observed in a myocardial infarction induced heart failure model in hamsters (226). Similarly, guinea pig models of heart failure induced by pressure overload also have increased levels of PKCα and PKCε (227). These findings showcase that Cx43 S368 phosphorylation regulated by PKCs may serve as a therapeutic target in arrhythmias and heart failure.

## PKC/Cx43 pS368 circuit in therapy against cardiac diseases

Peptides that target Cx43 have been extensively studied for their pharmacological benefits in cardiac diseases. These peptides mimic the extracellular loop of Cx43 or the cytoplasmic C terminal domain. For instance, Gap26 and Gap27 are based on the extracellular domains of Cx43 while Gap19 and αCT1 include amino acids of the C terminal tail of Cx43 (211). αCT1, or alpha Connexin carboxy terminus 1, has been tested *in vitro* and *in vivo* for its effect against ischemia/reperfusion injury. It was designed to inhibit the interaction of Cx43 with Zona Occludens-1 (ZO-1). Cx43 binds with the postsynaptic density/disks-large/ZO-1 (PDZ2) domain of ZO-1 which results in the remodeling of gap junction plaques. This interaction has been reported to increase cardiomyopathies and heart failure in humans (228). In mouse models of cardiac injury, αCT1 was shown to interact with the PDZ domain of ZO-1 and induce phosphorylation of Cx43 at S368 which can reduce intercellular communication through Cx43. Cryoinjury CD1 mice treated with αCT1 display an increase in Cx43 plaques in the intercalated discs while control-treated groups have a higher level of Cx43 remodeling from plaques to lateralized distributions. αCT1 treatment promoted the level of Cx43 S368 phosphorylation in the plaques in a PKCε dependent manner (178). Furthermore, αCT1 can protect hearts from ischemic injury and preserve ventricular function likely due to its interaction with the Cx43 C-terminal helix 2 domain and not due to its interaction with the PDZ domain of ZO-1. αCT1 by itself leads to an increase in Cx43 S368 phosphorylation in a concentration-dependent manner (229). *In vivo* studies confirm that αCT1 reduces arrhythmia in cryo-injured hearts and increases the rate of depolarization (178, 228). αCT1 has reached Phase III clinical evaluation for healing chronic skin wounds associated with non-cardiac diseases such as cancer and ulcers in which Cx43 plays a prominent role (230, 231). Phase I and Phase II clinical trials with αCT1 in venous leg ulcers, diabetic foot ulcers, and cutaneous scarring/Laparoscopic incisions showed enhanced wound closure as compared to control-treated patients. However, the phase III clinical trial with αCT1 was terminated although no adverse toxicity was reported (232). Since αCT1 has also shown promising results *in vivo* against arrhythmia and ischemia/reperfusion injury, clinical trials utilizing αCT1 or other agents to promote Cx43 S368 phosphorylation in cardiac diseases seem viable (178, 229).

Another method of increasing Cx43-S368 phosphorylation is by inducing PKCε activity that directly targets S368. Using ψεRACK, a PKCε specific activator, revealed that PKCε activation induces protection against ischemia/reperfusion and hypoxic injury. ψεRACK treatment increases PKCε translocation to the plasma membrane in isolated cardiomyocytes and in transgenic mouse hearts that express ψεRACK postnatally (233). This activation could be a therapeutic approach toward utilizing PKCε and Cx43 pS368 relationship against ischemia. Drugs that upregulate PKCε such as adenosine have been used as an adjunct to thrombolysis or percutaneous intervention to treat acute myocardial infarction in patients. A randomized study showed that adenosine-treated patients had reduced infarct size compared to that observed in the placebo group (234). Adenosine was administered intravenously to patients which were then followed for new incidences of congestive heart failure after 24 h, or subsequent hospitalization after congestive heart failure, or death due to any causes within 6 months. Although no differences between treated and placebo groups at each of these endpoints were observed, the study did find that higher doses of adenosine correlated with smaller median infarct size. Therefore, pharmacological strategies that promote PKC-mediated Cx43 S368 phosphorylation might be more fruitful based on the importance of this interaction in the heart.

Cx43 S368 phosphorylation is not always reduced in cardiac diseases. For instance, myocarditis and arrhythmia can lead to hyperphosphorylation of Cx43 (184). Despite the presence of various CX43 inhibitors such as carbenoxolone and peptide inhibitors such as Gap26, issues regarding specificity and off-target effects have remained. Interestingly, gap junction inhibitors that show specificity toward the Cx43 S368 phosphorylated confirmation have been generated, such as the lipidated connexin mimetic peptide SRPTEKT-Hdc. Treatment with this peptide leads to a reduction in dye coupling and Ca^2+^ wave propagation through Cx43. MDCK cells expressing either phosphodeficient or phosphomimetic mutants of Cx43 S368 were treated with SRPTEKT-Hdc (235). The potency of inhibition by SRPTEKT-Hdc was greater in the phosphomimetic group than in the phosphodeficient group suggesting that SRPTEKT-Hdc had specificity toward the phosphorylated form of Cx43 S368. The cardioprotective effect of this peptide is yet to be examined in cases where hyperphosphorylation of Cx43 S368 is observed. Reduction of Cx43 S368 phosphorylation can also be successfully achieved through the inhibition of PKC using PKC-specific inhibitors such as Calphostin C (108). Since heart failure shows an increase in PKCε activity, inhibition with PKCε specific inhibitor εV1-2 decreases symptoms associated with heart failure including parenchymal fibrosis and fractional shortening (236). However, the usage of PKC inhibitors should be examined in more depth since most PKC inhibitors affect several PKC isozymes when used at higher concentrations and potentially other kinases as well. Therefore, PKC inhibitors that target the PKC Cx43 S368 phosphorylation circuit should be explored further in cardiac defects as they may provide greater specificity.

## Conclusion

Various PKC isozymes directly target and phosphorylate S368 on the C terminal tail of Cx43 in cardiomyocytes. This acts to regulate gated conductance as well as a degradation signal for the Cx43 plaques to reduce gap junctional coupling in the cardiac system. Here we explored the prevalence of PKC isozymes along with the level of Cx43 S368 phosphorylation in different cardiac diseases (Table 2). Cx43 S368 phosphorylation occurs in healthy embryonic and adult hearts. However, the level of this phosphorylation varies in different cardiac disease states. Future *in vivo* studies in animal models and therapies based on this PKC/Cx43 pS368 relationship should consider this variation while designing drugs effective toward cardiac diseases.

**TABLE 2:** Summary table of the phosphorylation of Cx43-Serine-368 and expression level of PKC isozymes in various cardiac disorders.

Cardiac disease	Species/model	Cx43-serine-368 phosphorylation	PKC isozymes expression	References
Ischemia – reperfusion	Rat (Ischemia – reperfusion model)	↓ Ischemia ↑ Perfusion	↑ Perfusion PKC α and PKCε	(25, 175)
Myocarditis	Rat [experimental autoimmune myocarditis (EAM)]	↑	↑ PKC α	(184, 186)
Hypertrophy	Mice	–	↑ PKCβ	(189)
Hypertrophy	Neonatal cultured rat cardiomyocytes	–	↑ PKCα	(190)
Dilated cardiomyopathy	Human heart explants	–	↑ PKCα and PKCβ	(191)
Right ventricular hypertrophy	Rats	↓	–	(194)
Congestive heart failure	Dogs	↓	–	(205, 206)
Isoproterenol induced fibrillation	Rats	↑	↑PKCε	(225)
Myocardial infarction and heart failure	Hamsters	–	↑ PKC α, β, ε, ζ	(226)
Pressure overload induced heart failure	Guinea pigs	–	↑ PKCα and PKCε	(227)

## Author contributions

RP wrote the initial draft. All authors edited and provided further input in the manuscript preparation.
